# Noise Maps for Quantitative and Clinical Severity Towards Long-Term ECG Monitoring

**DOI:** 10.3390/s17112448

**Published:** 2017-10-25

**Authors:** Estrella Everss-Villalba, Francisco Manuel Melgarejo-Meseguer, Manuel Blanco-Velasco, Francisco Javier Gimeno-Blanes, Salvador Sala-Pla, José Luis Rojo-Álvarez, Arcadi García-Alberola

**Affiliations:** 1 Cardiology Service, Arrhythmia Unit, Hospital General Universitario Virgen de la Arrixaca, El Palmar, Murcia 30120, Spain; estrella.everss@urjc.es (E.E.-V.); francisco.melgarejo@goumh.umh.es (F.M.M.-M.); 2 Department of Signal Theory and Communications, University of de Alcalá, Alcalá de Henares, Madrid 28805, Spain; manuel.blanco@uah.es; 3 Department of Signal Theory and Communications, Miguel Hernández University, Elche, Alicante 03202, Spain; javier.gimeno@umh.es; 4 Instituto de Neurociencias, Miguel Hernández University–CSIC, Alicante 03550, Spain; salvador.sala@umh.es; 5 Department of Signal Theory and Communications, Rey Juan Carlos University, Fuenlabrada, Madrid 28943, Spain; joseluis.rojo@urjc.es; 6 Center for Computational Simulation, Universidad Politécnica de Madrid, Boadilla, Madrid 28223, Spain

**Keywords:** noise maps, ECG, noise clinical severity, Holter, external event recorder, long-term monitoring, noise bars

## Abstract

Noise and artifacts are inherent contaminating components and are particularly present in Holter electrocardiogram (ECG) monitoring. The presence of noise is even more significant in long-term monitoring (LTM) recordings, as these are collected for several days in patients following their daily activities; hence, strong artifact components can temporarily impair the clinical measurements from the LTM recordings. Traditionally, the noise presence has been dealt with as a problem of non-desirable component removal by means of several quantitative signal metrics such as the signal-to-noise ratio (SNR), but current systems do not provide any information about the true impact of noise on the ECG clinical evaluation. As a first step towards an alternative to classical approaches, this work assesses the ECG quality under the assumption that an ECG has good quality when it is clinically interpretable. Therefore, our hypotheses are that it is possible (a) to create a clinical severity score for the effect of the noise on the ECG, (b) to characterize its consistency in terms of its temporal and statistical distribution, and (c) to use it for signal quality evaluation in LTM scenarios. For this purpose, a database of external event recorder (EER) signals is assembled and labeled from a clinical point of view for its use as the gold standard of noise severity categorization. These devices are assumed to capture those signal segments more prone to be corrupted with noise during long-term periods. Then, the ECG noise is characterized through the comparison of these clinical severity criteria with conventional quantitative metrics taken from traditional noise-removal approaches, and noise maps are proposed as a novel representation tool to achieve this comparison. Our results showed that neither of the benchmarked quantitative noise measurement criteria represent an accurate enough estimation of the clinical severity of the noise. A case study of long-term ECG is reported, showing the statistical and temporal correspondences and properties with respect to EER signals used to create the gold standard for clinical noise. The proposed noise maps, together with the statistical consistency of the characterization of the noise clinical severity, paves the way towards forthcoming systems providing us with noise maps of the noise clinical severity, allowing the user to process different ECG segments with different techniques and in terms of different measured clinical parameters.

## 1. Introduction

The electrocardiogram (ECG) signal, which depicts the electrical activity of the heart, is an efficient tool to observe its health condition because it depicts useful electrical information on the cardiac system collected by noninvasive means using a set of electrodes attached to the surface of the human body [1]. Whereas the usual ECG registers the signal during short periods of a few seconds, Holter monitoring allows us to record the ECG for longer time intervals, typically of 24 h. Nowadays, it has become usual to use this in order to find anomalies that do not appear in short-term ECG, such as paroxysmal, self-limited arrhythmias presenting as episodes separated by hours or days [2]. It is also a very attractive method to provide health care solutions in combination with the information and communication technologies [3,4], including health monitoring in remote areas using mobile devices [5,6].

Currently, the interest in monitoring patients over several days is growing because of its potential to find anomalies that remain undetected in standard ECG and 24 h Holter. This kind of register is referred to as long-term monitoring (LTM) recordings, and these have been found to be useful for the detection of subclinical atrial fibrillation in patients with cryptogenic stroke [7,8], for the detection of non-sustained atrial or ventricular arrhythmias in patients with heart failure [9], and for the assessment of autonomic parameters obtained from heart rate variability analysis [10]. Holter ECG is obtained from portable devices while patients continue with their daily activities; thereafter, the signal is highly affected by noise and artifacts. Thus, as a result of the extensive use of Holter ECG, noise has become a matter of major concern, particularly in LTM recordings, because its presence may result in wrong diagnoses.

The main sources of noise are patient movements (baseline wander noise, electromyographic noise, and electrode motion), powerline or electronic-device interference at data collection, signal processing, or medical equipment [11]. Traditionally, dealing with poor-quality ECG has been faced as a denoising problem for which the target consists of improving some quality metrics, such as the root mean square (RMS) or the signal-to-noise ratio (SNR), which are often measured on artificially contaminated ECGs. Recent quantitative analysis has been performed within this framework using different signal processing techniques, such as transform domains [12,13,14,15,16], independent and principal component analysis [17,18], adaptive filtering [19], genetic algorithms [20], empirical mode decomposition [21,22], fuzzy logic [23], or neural networks [24,25,26].

The underlying assumption of these approaches is that the denoising process can provide a valid signal if the resulting metric values are solid enough. However, none of the used measurement parameters report information about diagnostic features allowing us to assess their clinical validity. For example, we note that sometimes, a moderate SNR does not affect the ECG morphology, whereas similar SNRs in different conditions may dramatically decrease the signal quality. Additionally, on some occasions, the ECG is so strongly altered that none of its waves are recognizable; thus they become useless. In consequence, rather than noise elimination, an alternative approach is followed in recent research focusing on two aspects, namely, noise quantification from a clinical standpoint, and ECG quality assessment. The quality is considered in this work as a synonym of clinical validity; that is, a signal is considered *good quality* when it can be used for clinical purposes. According to this view, the signal processing of LTM for quality ECG purposes would no longer be limited to artifact removal, but it should also include the identification of severely corrupted segments, which are clinically invalid with different severity levels, in order to avoid false diagnosis. One of the most clarifying contributions following this approach is given in [27], which proposes a method for classifying the quality of an ECG into five levels. The paper is also a good summary of previous works on this topic. Other examples of automatic detection and classification of noise are [28,29], although these two works do not rate the noise in the ECG from a clinical standpoint.

Therefore, and according to this novel approach, our hypothesis is that it is possible (a) to create a clinical severity score for the effect of noise on the ECG, (b) to characterize its consistency in terms of its temporal and statistical distribution, and (c) to use it for LTM evaluation of signal quality, providing a better criterion for diagnostic index extraction when compared to conventional quantitative noise magnitude measurements.

To test this hypothesis, we scrutinized the following elements. First, we proposed to create and use noise maps, defined as a simple overview of the temporal distribution of the proportion of noise with different quality throughout time windows with adequate duration for long recordings. Second, we propose to create a measurement of the noise clinical severity, according to the criteria of a specialist on the impact that different noise intensities and presence will have on subsequently measured clinical indices. This noise clinical severity will be potentially used as the gold standard for analyzing the conventional quantitative noise measurements. We subsequently analyzed the noise clinical severity measurement for long-term recordings from ECG external event recorders (EER) in 10 patients and in one case from 7 day Holter monitoring on a detailed timeline. A preliminary version of this work has been previously introduced [30].

The scheme of the paper is as follows. In Section 2, we describe our patient database and the LTM Holter patient case. The clinical severity criteria for ECG noise are also described, as well as the state-of-the-art proxies for quantitative noise and the definition of noise maps. The results are presented, and the achieved noise classification criteria are summarized in Section 3, together with the noise maps for clinical and quantitative noise criteria; the noise statistical distributions are scrutinized both in the EER database and in the LTM Holter case example. Some relevant considerations for the powerline noise and the inter-observer variability are also included therein. The discussion and conclusion of the present work are finally addressed in Section 4.

## 2. Materials and Methods

This section is structured as follows. Firstly, the recordings used to address the LTM analysis are presented in two scenarios. Secondly, the methodology followed for launching a clinical severity gold standard is summarized, and several quantitative metrics of noise are introduced. For all of these kinds of noise, their characterization is then addressed in terms of their time distributions, by defining the noise maps, and in terms of their amplitude distribution, by using their by-sample statistical distribution of the estimated noise.

### 2.1. Materials

The data used for this study were gathered from two different continuous-recording ECG sources, namely, an EER (Sorin SpiderFlash-t) and a 7 day Holter device (Delmar Reynolds Lifecard CF). Data were obtained from clinical indications from the Arrhythmia Department of the University Hospital Virgen de la Arrixaca at Murcia, Spain.

EER devices perform continuous ECG ambulatory monitoring, and they also analyze the signal in real time, looking for QRS complexes (corresponding to ventricular depolarization deflections). Arrhythmic event detection is based on the rate and regularity of the QRS series [31], but in practice, noise and artifacts also often trigger the event recording, even during sinus rhythm. Every detected event (arrhythmia, artifact, or noise) triggers an automatic recording with a duration of about 30 to 300 s, at a sampling frequency of 200 Hz. The system has three electrodes, yielding two signals for subsequent analysis. Our database consisted of data from 10 patients (5 women and 5 men, 65.20 ± 23.52 years) who had been referred to the hospital for palpitation, syncope, or presyncope evaluation.

The use of the EER recordings as a convenient support for LTM was decided upon after considerations by medical staff. From a clinical point of view, the interest of the short recordings of EER in this scenario is twofold. On the one hand, they are a real problem in clinics, as these devices are likely to be working intensely and used during the next years. On the other hand, they almost certainly select the most interesting segments of the continuous signal, either for being real arrhythmias or for being artifacts, that are considered as arrhythmias by the device. In the second case, these segments represent noise with similar properties to the arrhythmias, which is what we wish to classify correctly (for instance, 50 Hz noise and a constant line for amplifier saturation can be more readily detected, and these do not hold much clinical interest). Our view is that if we are able to discriminate well between the noise in these EER recordings, it will be easier to work with LTM in Holter recordings, which often includes less-noisy signal regions to the global patient map.

A continuous and exhaustive classification and labeling of noise severity in one 7 day Holter recording was also made. The mean left-ventricular ejection fraction (LVEF) in this database was 36.6 ± 9.6%; 86.8% of patients were in the New York Heart Association (NYHA) class I or II and their ages were 54.1 ± 13.9 years. All the records were continuously registered at a sampling frequency of 128 Hz. Noise clinical severity was evaluated in only 1 out of the 53 heart failure patients of the database because of the high complexity and time-consuming process of labeling (see [10] for more details). More than 10,000 screens from this recording were manually supervised and labeled by a trained expert for more than 150 h.

Whereas a single 7 day patient can be seen as limited and could have too patient-specific noise, this approach represents a trade-off between viability and scope of the present study. The time required for the continuous labeling of the 7 day Holter was about 150 h, which was around 20 days of continuous work only to label and effectively a longer period than this for a researcher workload. Hence, we considered that this first work was necessary, as far as that it would provide us with relevant considerations to be taken into account for effectively developing the gold standard. If we wish to expand the gold standard in the future to a wider set of 7 day Holter recordings, then it is necessary to have a starting point that is informative enough to later make it viable and efficient from a human resource viewpoint.

Nevertheless, as a comparison to the scope provided by the EER database, the MIT Noise Stress database [32,33] is often used as the gold standard in this environment (although with different purposes of algorithm tuning). This widely used database consists of about 6 h, with segments of 12 half hours in two patients (118 and 119) and artificially added noise with six different SNRs. In this work, only in EER did we build more than 6.5 h of detailed and continuously time labeled segments in 10 patients; hence both databases are comparable in terms of duration, and the present database has an improved scope in terms of representing different patients.

### 2.2. Noise Clinical Severity

This work deals with the quality of the ECG, under the assumption that good quality means clinical validity of the signal, whereas poor quality compromises the clinical value of the ECG for diagnosis support. According to this view, and rather than noise elimination, our attention here is devoted to noise quantification for the purpose of identifying clinically invalid segments to analyze ECG measurements on them. Therefore, the establishment of quality criteria with clinical validity is the first point to be tackled.

We defined and validated a set of noise severity criteria in real ECG signals obtained from EER devices in 10 patients (see [30] for details on the database). An expert cardiologist (A.G.A.) made a qualitative description of the noise levels in Holter recordings in terms of their impact on the clinical diagnosis of basic parameters, such as the ECG waveform and heart rate distortion. After several iterations, a trained expert (E.E.V.) manually labeled the ECGs from our patient database. We note that it would have been ideal to have had two observers for each of the recordings in order to generate the labels, but this represented a limitation in terms of workload. Nevertheless, a set of recordings was initially scrutinized with the use of two observers, and given that there was a high concordance between them, an agreement could be readily achieved by a set of rules after training the expert in a representative set of complicated examples; an iterative process was used to review the doubtful cases together.

After this long iterative process, we established and applied the following set of criteria to be used as labels in the ECG segments:

- Noise-free (type 0): segment without noise.
- Low noise (type 1): some noise present in the segment, but P and T waves (corresponding to atrial deporalization and ventricular repolarization, respectively) and the QRS complexes are readable and their morphology can be identified.
- Moderate-noise (type 2): noisy segment in which only the QRS complexes are reliably identified, in at least three consecutive beats.
- Hard-noise (type 3): noisy segment with hardly recognizable or unrecognizable QRS complexes.
- Other noise (type 4): segments are calibration pulses or straight lines because of the complete absence of signal or amplifier saturation.

It can be seen that these proposed noise criteria are based on the clinical impact of the noise on the parameters to be measured in the ECG, independently of power noise and signal magnitude descriptions. We noted that this final noise classification was closely related to those provided by other references (e.g., [27]). However, and in contrast to other works, our approach created a continuously running labeling for all the times of all the signals in our database. Therefore, after achieving uniform criteria and classifying all the EER segments in these types of noise, we obtained a running gold standard for noise taxonomy in terms of its clinical severity.

### 2.3. Noise Measurements using Quantitative Metrics

In order to scrutinize the impact of the noise on the ECG clinical interpretation, we also analyzed the relationship between noise intensity and clinical severity. The measure of the distortion in an ECG recording was undertaken here for three types of noise, namely, baseline wander (BW), powerline interference (PLI), and standard deviation noise (SDN). BW and PLI were chosen because they are the most usual types of noise in cardiac records, and they are also easy to extract from the ECG signals. In addition, SDN is a novel measure proposed in this work, which was designed to take into account events that make the signal unreadable, such as signal loss or gain saturation due to electrode disconnection.

Among the existing methods to quantify noise, the following implementations were used here:

1. BW was calculated by using a cubic spline with a third-order polynomial interpolation and a 0.8 s time window for node estimation.
2. The quantification of PLI was made with a notch filter with the center frequency at 50 Hz.
3. The proposed SDN was extracted by following these steps: (a) the standard deviation of the signal was computed in blocks of 0.5 s; (b) every 10 blocks, the mean and the standard deviation were calculated; (c) finally, the mean plus twice the standard deviation was used as a measure of the noise for each block.

**Figure 1:**
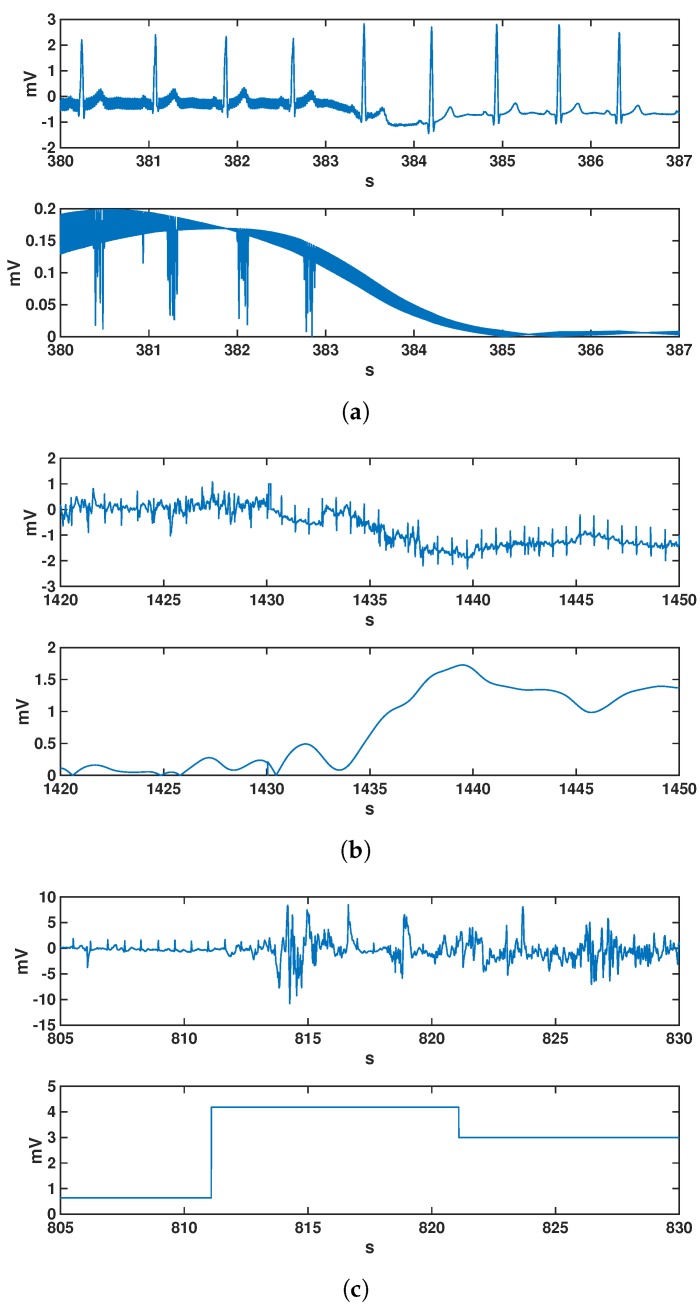
Illustrative examples of estimated noises for the different quantitative types analyzed in this work. Each panel (**a**–**c**) shows two sub-panels, one for the cardiac signal extracted from the event recorder, and another for the noise of different kinds and estimated from that same signal: (**a**) cardiac signal with significant presence of powerline interference (PLI) noise; (**b**) cardiac signal when relevant baseline wander (BW) noise is present; (**c**) cardiac signal when standard deviation noise (SDN) noise exhibits high values. Axis represent signal amplitude (vertical, in mV) vs. time (horizontal, in s).

Figure 1 shows several illustrative estimated examples of these noise types. We note that in general, these are not in fact independent from each other, as BW is partly included in the SDN calculation, and vice versa. In any case, we used these to scrutinize the different quantitative measurements in terms of the expected quality and to compare them with the noise clinical severity.

Once the measurements of noise intensity are available, either from the clinical severity criteria or from the three quantitative metrics, the ECG signal can be characterized in terms of its distortion. The noise characterization was tackled from two complementary viewpoints, namely, time distribution and amplitude distribution. The methods for the time and amplitude characterization are explained next.

### 2.4. Time Characterization with Noise Maps

We propose to tackle the characterization of the noise severity in the time domain by means of a new graphical representation, to be called noise maps. A noise map allows us to look at the quality of an ECG at a glance; thus we are able to scrutinize the extent to which the quality of the signal changes with time, and thus a noise map just depicts the noise level in a given ECG recording over time and for all the recording times.

Noise maps can be used when the noise intensity is determined either by clinical or quantitative criteria. In both, the following steps are followed to build a noise map from an ECG recording:

- First, the ECG signal is divided into segments, which are labeled according to their noise power; the labels for clinical severity were defined in Section 2.2.
- Afterwards, quantitative noise is split into four unevenly distributed levels, which in this work were adjusted in the event recorder noise distributions in order to match the quantitative noise map as closely as possible for an expert observer (FMM) to the clinical severity noise maps. This action generates a segmentation on the ECG, for which a list needs to be stored that includes the reference number of each segment, its starting and ending times, and its noise severity labels.
- Finally, label category changes are used for the time instants to define different-size segments corresponding to the set of samples with the same label.

As a result, a noise map is the depiction of these label segments as a function of continuous time. We note that, for the quantitative noise measurements, the instantaneous noise can be readily defined and used as the basis for the noise maps.

**Figure 2:**
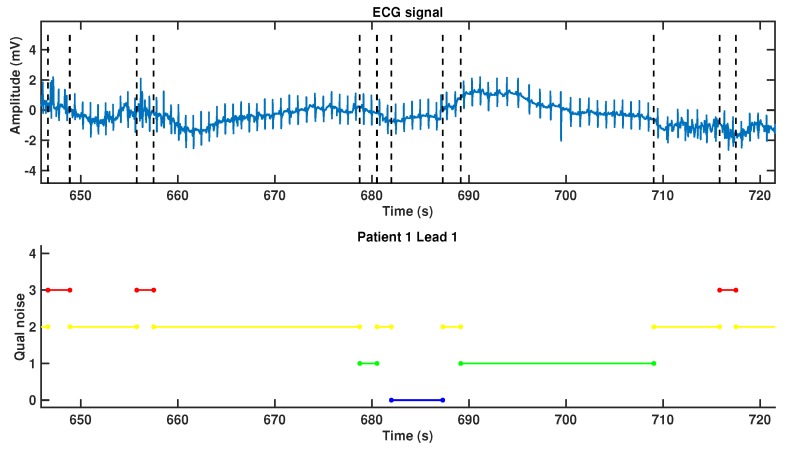
Noise map representation (bottom) of the clinical severity of noise observed in a 75 s electrocardiogram (ECG; top). Horizontal lines extend during the time periods for which each segment has been labeled by an expert. There are noise-free segments in blue (type 0) and segments in green, where the P and T waves, as well as the QRS complexes, are readable (type 1). In yellow segments, only the QRS complexes can be reliably identified (type 2), whereas segments with hardly recognizable QRS complexes, in red (type 3), are those for which no clinical parameter can be trustfully measured because of severe noise.

An example of a noise map can be seen in Figure 2, which corresponds to a 75 s ECG excerpt taken from an EER and labeled according to noise clinical severity criteria. Every heartbeat from the signal is tagged with a single label; hence, the label segments do not overlap each other. This representation reports reasonably well on the noise severity evolution over the recording. In this case, the severity ranges from noise-free (type 0) up to hard-noise (type 3). More than half of the ECG excerpt is labeled as type 2 (moderate-noise) or 3 (hard-noise). These segments stand for noisy blocks, which can scarcely be used for a reliable analysis of the full heartbeat. Therefore, and despite the short duration, the example corresponds to a noisy ECG when analyzed from a clinical viewpoint. This is consistent with the characteristics of the EER database because, as mentioned before, the result of an EER acquisition is the continuous recording of either arrhythmic or noise events, which can be likely labeled within some of the previously defined noise categories.

### 2.5. Statistical Characterization of the Noise Amplitude

Besides the representation of noise distribution over time, noise maps are also conceived to display coincidences between clinical severity and quantitative noise. Nonetheless, it may be considered that noise severity, when defined from a clinical point of view, can be somehow related to the quantitatively determined noise power. For this reason, the quality analysis of the ECG is next accomplished through the analysis of the naive amplitude, defined here as the absolute value of the estimated instantaneous noise.

Let $p\left(n\right)$ stand for the ECG noise probability density function (*pdf*), n(t), and let $p\left(n_{v}\right)$, $v=\left\{bw,pli,sdn\right\}$, be the specific noise *pdf’s* for the previously used quantitative components. We note that we assume that no further noise sources are present and that they are independent, which will not be true in general; nonetheless, these distributions are used here for descriptive purposes of different noise sources. Then, the following signal model is used:

(1) $$x\left(t\right)=x_{c}\left(t\right)+n_{bw}\left(t\right)+n_{pli}\left(t\right)+n_{sdn}\left(t\right)+e\left(t\right)$$

where both x(t) and $x_{c}\left(t\right)$ are the noisy and the noiseless ECG, respectively; $n_{v}\left(t\right)$, $v=\left\{bw,pli,sdn\right\}$ refers to the quantitative noisy components; and e(t) is an error term representing any other non-considered noise source that is still present in the signal. In order to further analyze the relationship between noise clinical severity and quantitative noise, we consider the conditional *pdf* with respect to the discrete levels of noise clinical severity, defined in Section 2.2 and denoted here as $l_{0},l_{1},l_{2},l_{3}$ and $l_{4}$. Hence, we can define the conditional *pdf* of each type of noise in terms of the noise clinical severity for their comparison, which, for the example of BW noise, is given by

(2) $$p\left(n_{bw}\right)=\sum_{i=0}^{4}p\left(n_{bw}|l_{i}\right)P_{bw}\left(l_{i}\right)$$

where $l_{i}$ denotes the type of noise clinical severity, $p(n_{bw}|l_{i})$ is the conditional distribution of BW noise with respect to type $l_{i}$, and $P_{bw}\left(l_{i}\right)$ is the prior probability of that type of noise in the BW noise component. These conditional *pdf’s* can be similarly established for the other noise components.

## 3. Results

### 3.1. Analysis of the Conditional Distributions

**Figure 3:**
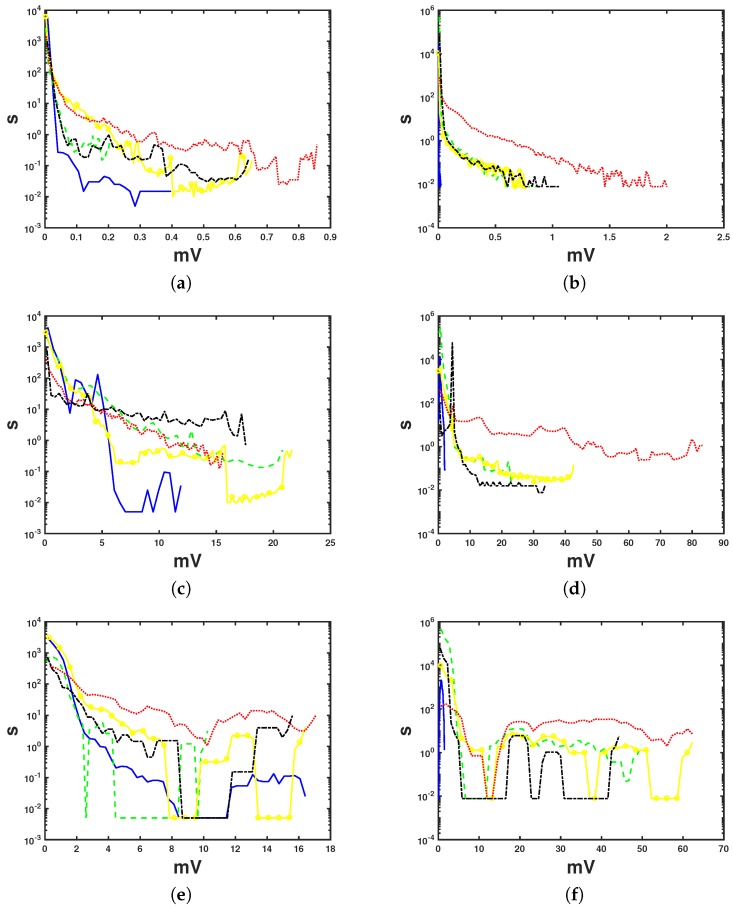
Estimated distributions (from scaled log-histograms) for the different noise types in external event recorder (EER) (**a**,**c**,**e**) and 7 day Holter (**b**,**d**,**f**) recordings. Noise samples (in duration, seconds, as multiplied times the sampling period) are represented in terms of the noise with the same voltage level (in mV). The color code is similar to that for the noise maps: noise-free segments—blue; low-noise—dashed, green; moderate-noise—crosses, yellow; hard-noise—dotted, red; and other noises—dash–dot, black. Axis represent number of samples scaled to their time duration (vertical, in s) vs. amplitude (horizontal, in mV).

The log-scaled histograms depicted in Figure 3 correspond to the distributions of the right-hand side in Equation (2), and each curve represents one level of noise clinical severity. Left (right) panels correspond to noise in EER (7 day Holter) recordings. From top to bottom, the distributions are shown for PLI noise in panels (a,b), for BW noise in panels (c,d), and for SDN noise in panels (e,f). Each plot represents the value of the quantitative noise conditionally separated according to the clinical severity label.

All the curves represent the full range of existing values for the noise in the horizontal axis; thus, we may see that PLI noise (upper plots) for noise-free segments has a distribution confined to 0.4 mV in EER and to 1 mV in 7 day Holter. In both cases, the amplitude distributions for different levels overlap one another; hence, the PLI amplitude alone does not yield a measurement of the noise clinical severity. A similar result is obtained for BW noise (middle plots). The distributions of Figure 3c exhibit heavier tails than the previous distributions, which makes it evident that high values of BW noise may not correspond with hard-noise in terms of clinical severity. Distributions for 7 day Holter (Figure 3d) are also heavy tailed and fairly similar to those of EER. The heaviest-tailed distribution is the hard-noise distribution in 7 day Holter, which is almost uniform. This fact again prevents us from making a division in terms of clinical severity just by using the BW noise amplitude as a criterion.

A similar situation occurs again with SDN noise for EER (Figure 3e), where the hard-noise covers the full voltage range, hence avoiding the labeling according to clinical severity criteria. Regarding the distributions of the remaining noises, these are lower in amplitude and are concentrated in specific regions, exhibiting blank spaces (values in the vertical axis lower than 100 μV) in between. In the 7 day Holter case (Figure 3f), the noise distributions coexist in an interval that goes up to the local minimum for the hard-noise distribution (around 12 mV). This overlapping means again that small variations in the SDN noise can be labeled in different clinical severity categories. After this minimum, all the distributions behave similarly to those in Figure 3e, with the aforementioned incapability of labeling the signal according to the clinical severity criteria based on SND noise. We note that the differences in the noise amplitude of the horizontal axis for distributions of the same class of noise are due to the different nature of the data and that normal ECG segments seldom appear in EER records because they capture noisy events, whereas many normal sinus ECGs can be often found in the 7 day Holter record.

### 3.2. Results of Noise Maps for EER

Table 1 shows the durations of the different clinical severity noises for each patient in both leads of the EER and according to their clinical severity, which overall were 3 h 27 m 56 s (noise-free, 26.77%), 3 h 1 m 15 s (low-noise, 25.67%), 4 h 4 m 50 s (moderate-noise, 31.52%), 1 h 29 m 18 s (hard-noise, 11.50%), and 53 m 27 s (other noise, 6.88%).

Given that the quantitative noise amplitude is given as a continuous-variable function, a set of labels must be determined in order to present these as noise maps. We used labels to represent noise-free, as well as low-, moderate-, and hard-noise segments. To obtain each category, a set of different thresholds was manually set by using the amplitude histograms for all the quantitative noise. In order to set the different thresholds, the following rules were used: (a) the noise-free threshold was set in order to contain the near-zero values, (b) the hard-noise threshold was set to isolate the heavy tails of the distribution, and (c) the threshold to separate the low-noise and the medium-noise was set in about the middle of the regions delimited by the noise-free and hard-noise thresholds.

**Figure 4:**
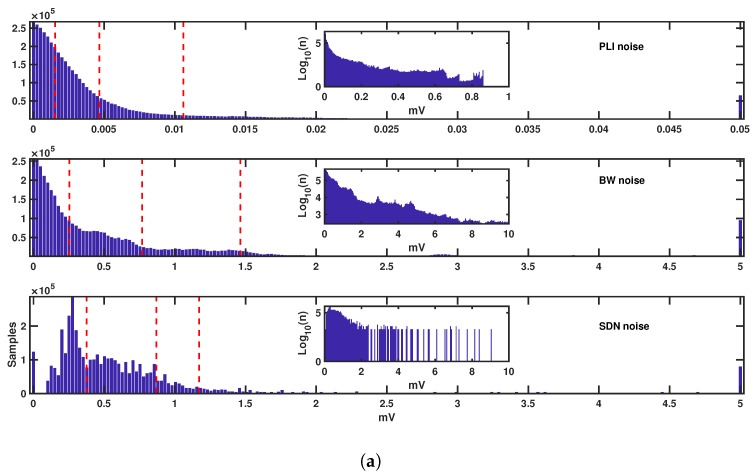

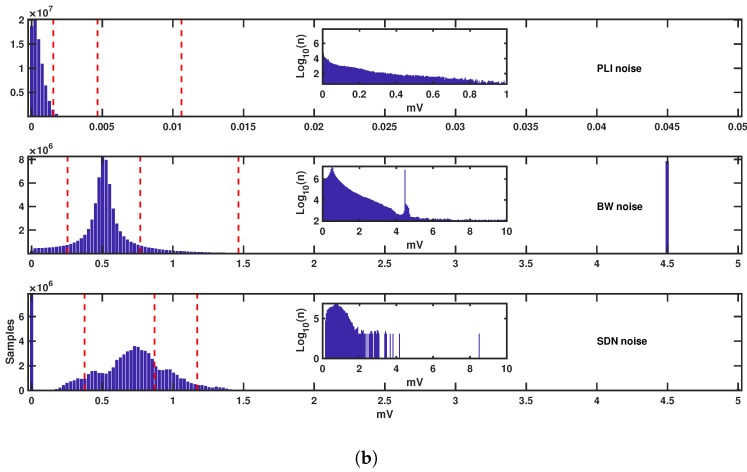
Histograms and thresholds established for the different quantitative noise types present in external event recorder (EER) (**a**) and 7 day Holter (**b**) recordings. Horizontal axis is the noise amplitude (in mV) as explained in Section 2.3, and vertical axis represents the number of samples per voltage bin. We note that the tails are better visualized in the logarithmic scale for the histogram of sample counts (insider plots in each panel). Axis represent number of samples (vertical) vs. their amplitude (horizontal, in mV).

The threshold divisions, as well as the histograms of each kind of noise, are shown in Figure 4. In order to set the threshold values, only the EER histograms were used, because these provided us with a wider view of the noise, as the records came from different patients and covered very different situations related to noise. We note that the horizontal axis has a much lower magnitude in the PLI noise, as far as that its amplitude is in general much lesser than that of BW and SDN noise. However, in all of these, the distributions are shown to be mostly exponential-like (PLI and BW noise) and with heavy tails, although SDN is slightly different and centered. The thresholds were chosen on the histogram regions, aiming to separate the low-amplitude region (close to zero amplitude), the heavy-tail region (large amplitude values), and different trends in the distribution mass morphology to separate low from medium regions.

**Figure 5:**
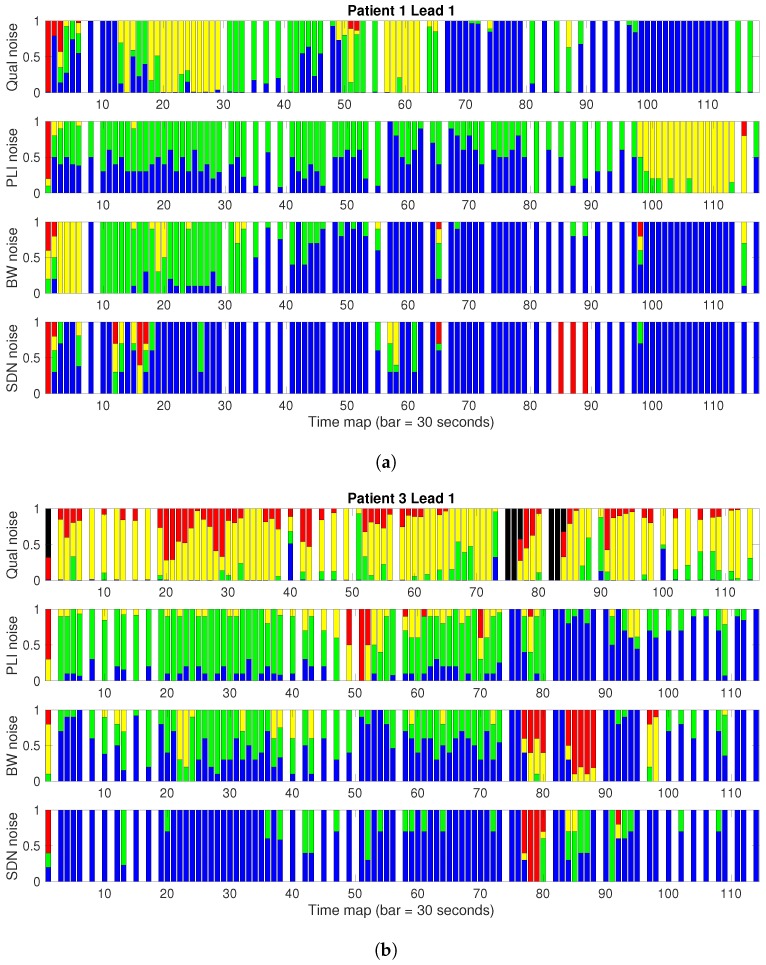
Example of external event recorder (EER) noise bars to compare qualitative and quantitative noise. Each bar represents 30 s segments of the recording, for patient 1 (**a**) and patient 3 (**b**).

The visualization of noise maps in long recordings can be operatively limited; hence, we also introduced the use of *noise bars*, as seen in the example in Figure 5. Noise bars were obtained by aggregating the durations of the same types of noise in fixed-duration segments, known as time bars, and then normalizing these in each time bar. We note that the time bar was 30 s in length for EER patients. Thus, a vertical bar stands for one single time segment, which shows the ratio of each kind of noise present throughout that time period. The same color code is used so that the lower (upper) part of the bar conveys the lower (harder) noise levels. This provides a profile view, giving a good idea of how clean or noisy a recording is and how many and which clinical parameters are possible to measure through it. Different time segments are separated by a blank bar for the case of EER when stored segments are not continuous in time.

Figure 5 shows the noise bars of patients 1 and 3, for noise clinical severity in terms of PLI, BW, and SDN (from top to bottom). In patient 1, according to the noise clinical severity (upper plot), all the clinical measurements are allowable (noise-free, in blue) except for a small section at the beginning (in red, 2 or 3 bars) and another predominantly yellow section (15 to 20 and 65 to 80 bars), where only QRS are morphologically recognizable. In contrast to the gold standard, if the noise analysis were based only on PLI (second plot), an area defined to be allowable according to the clinical standard would have been labeled as useless for measuring morphological parameters (yellow section). If we only look at the BW map (third plot), the first section would have been tagged as non-usable, which is not true according to clinical severity. The rest of the recording would have been identified as valid, which would have caused a wrong parameter measurement in those areas for which the clinical severity map is mainly labeled in yellow. If we analyze the quality according to the SDN noise map (lower plot), most of the recording would be valid, except for a couple of small areas tagged in red. However, these unacceptable areas in terms of quality can indeed be used to measure the morphological parameters. Given these differences, none of the combination of the three methods provides the definition produced by the gold standard.

Similar observations can be stated regarding patient 3. In this case, the gold standard advises us of a more distorted ECG than that of patient 1. For example, the noise maps from SDN and BW inform incorrectly that the signal has long noiseless periods (type 0, in blue), whereas the clinical severity map indicates that only the QRS complex is morphologically recognizable (type 1, in green).

**Figure 6:**
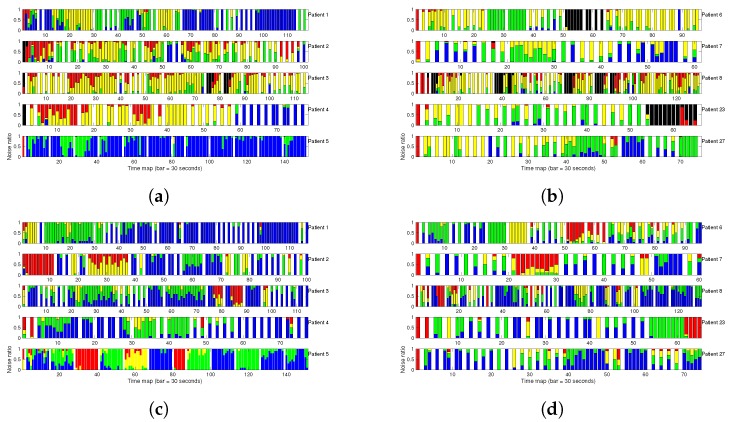

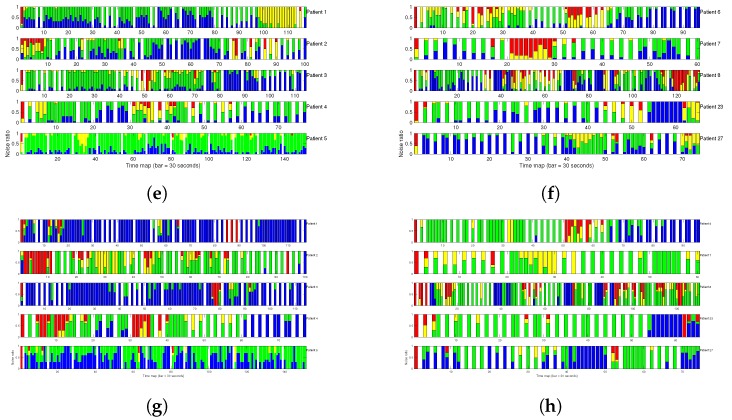
Noise bars of all the analyzed noise types for every patient in the external event recorder (EER) database: (**a**,**b**) noise clinical severity according to the gold standard; (**c**,**d**) baseline wander (BW) noise component; (**e**,**f**) powerline interference (PLI) noise component; and (**g**,**h**) standard deviation noise (SDN) component. Blank bars indicate those cases for which the stored EER segments are not continuous in time.

Figure 6 shows the noise bars for all of the eight EER patients with the four noise types, allowing us to scrutinize the correspondences among different noise types in this patient population. Panel (a) shows the gold standard noise bars for each patient, and these allow us to see that some recordings have very good quality; hence, these provide an excellent basis for measuring both waveform parameters and rhythm parameters based on the cardiac cycle, particularly in patients 1 and 5, for whom mostly green and blue bars are predominant. We also have questionable quality recordings for patients 2, 4, and 8, for whom red and black bars are predominant; hence measured parameters in these recordings should be either discarded or dealt with using extreme caution. Finally, we were able to measure the cardiac rhythm most of the time in patients 3, 6, and 7 (mostly yellow and green bars), but in these cases, morphological parameters were either avoided or dealt with using caution. Blank bars indicate the cases for which the stored EER segments were not continuous in time, given that these recordings were triggered either by arrhythmic events or noise artifacts, whereas in 7 day Holter recordings, pauses were often recorded through the isoelectric line and can be seen mostly in black bars.

Panel (b) shows the noise bars according to the BW noise, and its concordance with panel (a) is low. For instance, most of the patients have a stronger presence of blue and green bars, which should promote the calculation of all the clinical parameters in all the patients and is misleading. Similar conclusions could be drawn in panels (c) and (d) for PLI noise and SDN noise. We note also that, for the three quantitative cases, regions identified as non-suitable for measuring clinical parameters (mostly red bars) are generally different from each other, and are mostly different from the gold standard.

### 3.3. Specific Considerations of PLI Noise

As seen in Figure 4, the histograms of the estimated PLI noise could give the reader the idea of extremely low-noise power being present in the present database. We would like to emphasize that the method used for estimating the PLI noise essentially consisted of a notch filter and the subtraction of the filtered signal from the unfiltered signal. This means that, when no PLI noise was present, an extremely small residual was extracted, and if the recordings had long PLI-noise-free periods, this would represent noticeable density mass with low-noise amplitudes. Moreover, this PLI simple estimation method is sensitive to wide-band artifacts, such as electrode disconnection, saturation, and others. However, it would suffice to include here a quantitative estimation of PLI presence in the recordings for the present work, focusing on clinical severity of the noise.

**Figure 7:**
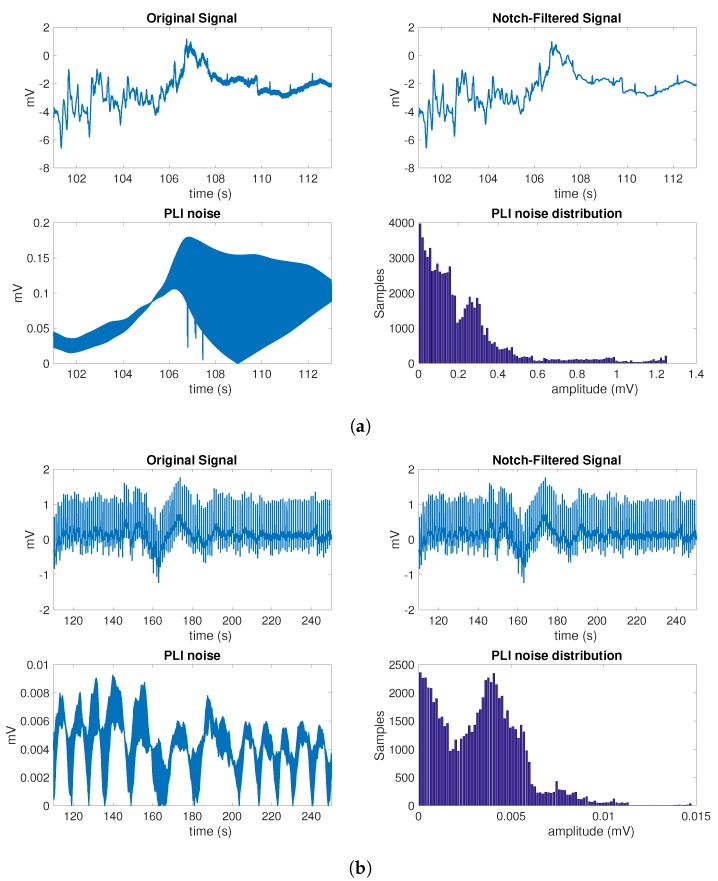
Additional considerations of the estimated powerline interference (PLI) noise: (**a**) example of external event recorder (EER) segment with high presence of PLI noise; (**b**) example of EER segment with all kinds of quantitative noise.

Figure 7 shows two signal examples, both on the dependence of the estimated PLI noise with other noise sources, and on the amplitude histogram and the relevance of its tails when the noise is not present throughout the signal. The difference between a high- and a low-PLI-contaminated segment can be observed in panel (a), where lower noise is present in the estimated 50 Hz noise in the first half but is due to artifacts, and much clearer noise is present in the second half, which is due to the true presence of 50 Hz noise. In panel (b), there is no 50 Hz noise present, so that the estimated PLI noise exhibits a low amplitude, and in this case, the histogram has intense mass density in very low noise amplitudes. From observing these partial-signal amplitude histograms, one can note that the significant information in this kind of noise is found in the tails, and that the cleaner the database, the more the pdf mass will concentrate on extremely low amplitudes. This is one of the reasons for representing the overall pdf with a logarithmic scale in the preceding figures.

We note also that the thresholds that are used in this work are not set intending to be universally established, but instead, we simply wished to show a quantification of the PLI noise, together with the other noises, and use these in the available recordings to scrutinize their correspondence with the clinical severity criterion. The use of other thresholds besides those herein would be recommended in terms of different applications. Nevertheless, after noting that there was not a great amount of PLI noise in the used recordings, we analyzed two new cases of EER (labeled here as patients 23 and 27), which were selected according to a noticeable presence of their PLI noise. Their noise bars can be seen in Figure 6 and are compared with the other cases; it could be confirmed that the PLI noise was represented by existing records in the previous dataset.

### 3.4. Considerations of Inter-Observer Variability

Aiming to have some information on the variability of the inter-observer criteria, we obtained the concordance of the two newly available recordings used in the preceding subsection. Another author (FMM) was trained to follow the designed criteria and labeled patients 23 and 27 according to these. Whereas this represents an *a posteriori* analysis of the inter-observer variability, it still brought into scene some relevant considerations.

Table 2 shows the confusion matrix for the concordance between both labelers (only for lead 1) in these recordings. We can see that types 3 and 4 are extremely concordant, whereas types 0–2 are less concordant; this is an expected result because of the partly subjective nature of the labeling. We note that the greater differences take place with neighbor severity types, a fact that was previously observed by other authors. For instance, a similar observation in [27] was solved by accounting for neighbor classes in the concordance measurement. We preferred here to retain the established classification and note that the concordance is in general high, as quantified by the Cohen’s kappa coefficient of 0.6987 [34]. We further note that types 0 and 1 (no noise and low-level noise) could even be put together in the same class, as they both yield good quality clinical indices with either no processing or with very light processing. In this case, and for informative purposes only, the confusion matrix is given in the same table when we put together types 0 and 1, and the Cohen’s kappa coefficient is raised up to 0.7217. This last result implies that, given the cost of the continuous labeling, in combination with the impact of considering types 0 and 1 as separate on the measurement, it could be recommended to put together these two classes from a practical point of view.

### 3.5. Results of the 7 Day Holter Case

In the 7-day Holter recording, the following amounts of noise were found (Table 1): 9 h 42 m 35 s (noise-free, 5.76%), 5 d 18 h 54 m 2 s (low-noise, 82.42%), 2 h 52 m 35 s (moderate-noise, 1.71%), 20 m 52 s (hard-noise, 0.2%) and 16 h, 41 m 52 s (other noise, 9.91%). Differences were observed in the shape of their histograms (Figure 4. For instance, there was less PLI noise in the 7 day Holter case (much narrower histogram). In addition, the BW and SDN distributions, which had their largest probability close to zero in the EEG, had close to a non-zero mean value in the 7 day Holter. This was checked to have happened because in this single case, the noise level often remained at a quasi-constant level. Even so, the widths of the histograms were qualitatively comparable, although with a lower tail for extreme amplitudes in both types of noise in the Holter case. Nevertheless, the same thresholds obtained from the EER recordings were used for the 7 day Holter case, as the former represented a wider population, to avoid overfitting to one single histogram.

**Figure 8:**
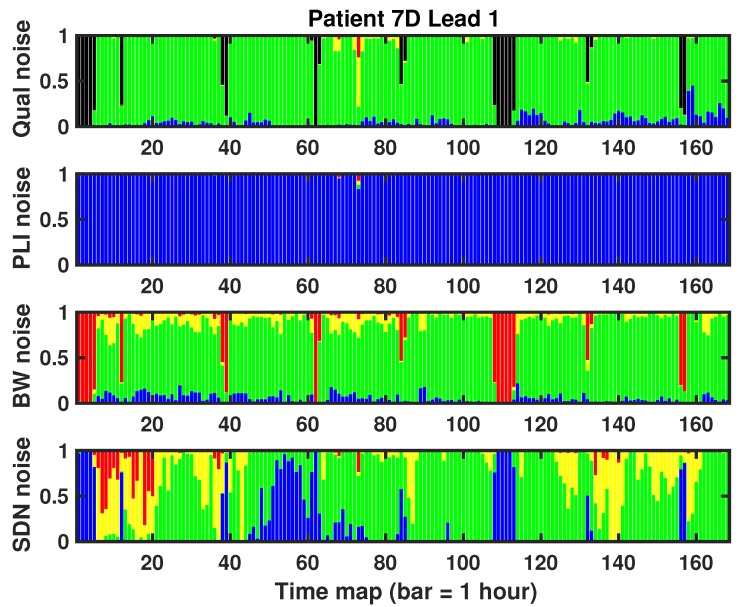
Example of noise bars for one lead of the 7 day Holter case. Each bar represents 1 h of recording (total of about 170 h) .

Figure 8 shows the noise maps for all the noise from this LTM recording. A comparison between quantitative noise bars and the gold standard shows that we could extract morphological parameters and cycle with clinical reliability in virtually all of the recording, except for the black sections, which correspond to disconnections and recharges during day 7. Compared with the PLI noise map, the whole record is mostly free of network noise, just as we saw in the first histogram of Figure 4b, for which, in this record, there was hardly any noise of this type. In the case of BW, the noise maps are overall in compliance with the gold standard regarding the low noise level (green), indicating that all the clinical measurements could be mostly taken. Nevertheless, we could discard a good number of sections (in yellow) of morphological parameters, without the need for this. The SDN noise map shows an extremely changeable behavior. It incorrectly marked valid black regions (those of the beginning), and it also incorrectly labeled as invalid (in red) at least three large regions. Accepting that in this case, the BW could be a relatively acceptable proxy to the gold standard, the populational characteristics of the EER recordings suggest that a wider population in 7 day Holter recordings exhibits larger dispersion and discordance, even for BW color bars.

**Figure 9:**
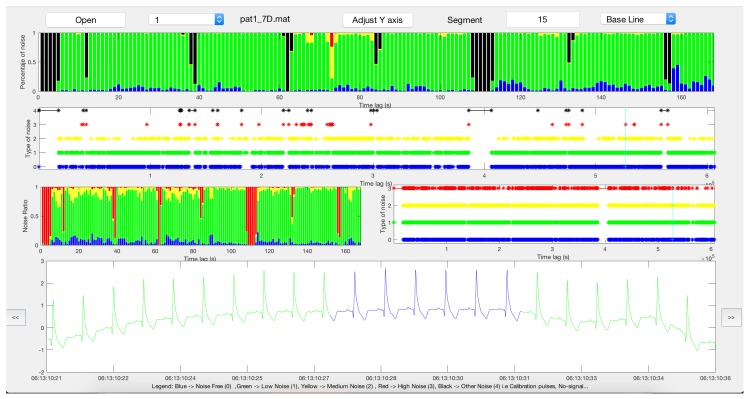
Noise maps’ interface of the 7 day Holter recording (lead 1). See text for details.

Finally, Figure 9 shows the front-end of our proposed noise maps when used with the 7 day Holter. The first panel shows the noise bars for the entire Holter, and the segments of different severity according to the gold standard are shown in the second panel. A high presence of low noise in more than 80% of the recording (in green) allows to measure all the clinical measurements, both in morphology and rhythm. The third row with two plots shows noise bars and their corresponding noise maps to measure the amount of BW, PLI or SDN components, so that qualitative versus quantitative comparisons can be performed to show the significance of the ECG clinical distortion. The lower panel shows the color-labeled ECG segment, which can be navigated on the second row and adjusted in time length.

## 4. Discussion

In this work, attention has been paid to noise quantification with the purpose of identifying clinically invalid ECG segments rather than noisy segment elimination, in terms of the clinical parameters (morphology and rhythm) that can be reliably measured from them. For this purpose, we first proposed a four-type criterion of noise clinical severity. The concept of noise maps has been defined as the representation of the temporal distribution of the noise proportion with qualitative levels, as well as with other discrete magnitude noise severity levels as assigned for different quantitative types of noise. Whereas the labeling process has been extremely time-consuming, the proof of concept shows that the noise maps with the clinical criteria show consistency with time and with statistical amplitude distributions. Hence, this work presents a solid basis, to be extended in the future for training intelligent systems capable of automatically determining the noise level severity from a complete ECG recording.

Our proposal is different from other noise descriptions based only on the level of the SNR estimated by mathematical metrics. When ECG signals are filtered to eliminate noise components (e.g., low and high frequencies), the ECG morphology-based magnitudes, which are usually useful for detecting cardiac pathologies, could be modified or distorted by these filters. Most of previous efforts in the ECG-noise study domain focus on the mitigation or removal of the noise in ECG recordings from public databases, for example, MIT-BIH Arrhythmia or Noise Stress Test databases. Differently from our present research, these database recordings are not continuously labeled in terms of their signal quality. In addition, these studies seldom describe a detailed noise scale classification, but only the usual BW, muscle artifact, and PLI noise powers [35,36,37,38], and controlled noise with different SNR values is often added to the ECG signal [39,40,41] or is assigned in global terms of acceptable or unacceptable signals for the ECG quality assessment [29,42,43].

To our best knowledge, the concept of noise maps with noise severity criteria has never been raised. In the scarcity of published evidence to support noise clinical severity descriptions, methods are mainly designed with the purpose of testing the denoising algorithms proposed by their authors. For example, the MIT-BIH Arrhythmia database was used in [27] to train a support vector machine classifier to perform quality classification of clean ECG signals with added noise of three different kinds, namely, muscle artifacts, BW interference, and electrode motion, with different SNR levels. Similarly to our work, they identified four types of quality level noise (minor, moderate, severe, and extreme). In [44], the cardiologists manually re-annotated the quality of 1500 10-s recordings and 25 60-min recordings, and they identified three types of noise from an energy point of view, namely, low-, medium-, and high-energy noise. In [40], six noise corruption levels were established, from no-noise to noise-positive detection, according to ECG amplitudes and slopes in different frequency bands). In [45], the authors described a mathematical algorithm for noise level estimation based on thresholds and comparison with R-waves. None of these studies scrutinized the statistical and temporal distribution of a clinically established gold standard for the noise severity.

It has been pointed out previously that noise components in one application could be interpreted as ECG signals in others, so that SNR can be an inexact measurement for clinical quality [46]. Although several studies have been published with 24 h Holter recordings, only some segments of a few minutes are considered to test different algorithms, and few detailed noise descriptions can be found [35]. With the recent increase in wireless mobile wearables and LTM clinical devices, the necessity of analyzing continuous segments is today even more evident [4]. Our results show that this is possible from a statistical and time-evolution point of view.

*Study limitations.* In this work, we have made an effort to assemble a database with continuous and detailed labeling. This specification can be difficult to merge with LTM, as intensive labeling over 7 day Holter turned into an extremely time-consuming task, as pointed in the methods section. We aimed to overcome this limitation by working on EER, which still was a time-consuming task, but to a lesser extent. The workload required to obtain a continuous labeling was one of the major challenges that we faced throughout the process. Nevertheless, it allowed us to create a reasonably wide database from which to start, a set of tools, and the interdisciplinary workflow to expand it. Future works will be devoted to using this high-quality and continuous database as the base for larger studies, likely by using machine learning techniques and semi-supervised working flows, such as those provided by incremental learning techniques.

## 5. Conclusions

ECG Holter recordings are commonly corrupted by noise. If ECG signals are filtered to eliminate these noise components, the ECG morphology and rhythm measurements can be modified and may distort accurate clinical classification. We propose a measurement of the noise clinical severity (noise maps) to be used for analyzing conventional quantitative noise measurements. In contrast to other studies, which add artificial noise to a clean baseline ECG, our approach used a real database with real noise (a single 7 day Holter and 10 EER recordings), and both the proposed criteria and the noise maps were based on the clinical needs for ECG interpretation. We conclude that their use is a highly desirable direction to improve the data quality when analyzing the measurements of long-term scenarios, such as EER recordings or 7 day Holter monitoring. With the relevance of the emerging modalities of LTM in current health and wellness scenarios nowadays, this represents a current necessity.

## Figures and Tables

**Table 1:** Duration (s) of noise types in leads 1 (left) and 2 (right) for external event recorder (EER; 1–8, 23 and 27) and 7 day Holter (7d) recordings.

N. Pat	Total Duration	Free	Low	Moderate	Hard	Other	Free	Low	Moderate	Hard	Other
1	2843,00	1383,55	774,36	621,97	57,26	5,88	1563,73	984,01	244,52	44,87	5,88
2	2433,00	195,11	719,02	994,89	480,24	43,76	180,68	725,48	994,67	488,42	43,76
3	2854,00	52,53	307,62	1891,07	434,43	168,36	32,50	287,83	1483,24	810,95	239,49
4	1673,00	338,98	44,54	861,47	392,53	35,49	345,50	31,84	514,52	745,66	35,49
5	4214,07	3451,95	709,36	10,19	29,76	12,82	3268,71	892,67	10,19	29,76	12,75
6	2134,00	65,05	611,16	1025,32	50,13	382,35	49,65	831,60	822,24	51,24	379,28
7	1172,00	376,00	356,04	394,61	37,70	7,67	403,24	364,32	357,38	39,41	7,67
8	3114,00	77,08	366,84	1561,03	512,31	596,76	50,16	341,99	1240,41	884,84	596,61
23	1278,00	31,38	501,94	347,98	86,20	310,51	31,38	501,94	347,98	86,20	310,51
27	1588,00	289,45	761,25	483,28	48,16	5,88	289,45	761,25	483,28	48,16	5,88
7d	606716	33602,99	500037,07	10355,21	1252,02	61468,7	36306,41	500046,92	10355,13	1252,02	58755,52

N. Pat: denotes number of patient.

**Table 2:** Confusion matrices, in minutes, for Cohen’s kappa coefficient in segments for external event recorder (EER) patients 23 and 27, for the established classification of clinical noise intensity (up) and after merging noise-free and low-noise classes (type 0 and 1).

**Observer 2**						
		**Free**	**Low**	**Moderate**	**Hard**	**Others**
	Free	3.67	1.86	0.00	0.00	0.00
	Low	0.30	20.46	0.29	0.00	0.00
Observer 1	Moderate	0.05	6.92	6.88	0.01	0.00
	Hard	0.00	0.03	0.06	2.14	0.01
	Others	0.00	0.00	0.00	0.00	5.08
**Observer 2**						
		**Free-Low**		**Moderate**	**Hard**	**Others**
Observer 1	Free–Low	26.30		0.29	0.00	0.00
	Moderate	6.97		6.88	0.01	0.00
	Hard	0.03		0.06	2.14	0.01
	Others	0.00		0.00	0.00	5.08
